# National survey of prevention and management of CMV infection in pediatric kidney transplantation in comparison to clinical practice guidelines

**DOI:** 10.3389/fped.2022.1057352

**Published:** 2022-12-16

**Authors:** Iona Madden, Véronique Baudouin, Marina Charbit, Bruno Ranchin, Gwenaëlle Roussey, Robert Novo, Florentine Garaix, Stéphane Decramer, Marc Fila, Elodie Merieau, Isabelle Vrillon, Ariane Zaloszyc, Julien Hogan, Jérôme Harambat

**Affiliations:** ^1^ Pediatric Nephrology Unit, Bordeaux University Hospital, Bordeaux, France; ^2^ Pediatric Nephrology Unit, Robert Debré Hospital, APHP, Paris, France; ^3^ Pediatric Nephrology Unit, Hôpital Necker Enfants Malades, APHP, Paris, France; ^4^ Pediatric Nephrology Unit, Hospices Civils de Lyon, Lyon, France; ^5^ Pediatric Nephrology Unit, Nantes University Hospital, Nantes, France; ^6^ Pediatric Nephrology Unit, Lille University Hospital, Lille, France; ^7^ Pediatric Nephrology Unit, AP-Hôpitaux de Marseille, Marseille, France; ^8^ Pediatric Nephrology Unit, Toulouse University Hospital, Toulouse, France; ^9^ Pediatric Nephrology Unit, Montpellier University Hospital, Montpellier, France; ^10^ Pediatric Nephrology Unit, Tours University Hospital, Tours, France; ^11^ Pediatric Nephrology Unit, Nancy University Hospital, Nancy, France; ^12^ Pediatric Nephrology Unit, Strasbourg University Hospital, Strasbourg, France

**Keywords:** cytomegalovirus, pediatric kidney transplant, prophylaxis, survey, valganciclovir

## Abstract

**Background:**

Cytomegalovirus (CMV) is one of the most frequent opportunistic infections in kidney transplant (KT) recipients and is a risk factor for patient and graft survival after KT. Center-to-center variation, optimal prevention and treatment strategies in pediatric KT are currently unknown. This survey aimed to assess current CMV prevention and treatment strategies used among French pediatric KT centers.

**Methods:**

A web-based survey was sent to all 13 French pediatric kidney transplantation centers.

**Results:**

Twelve (92%) centers responded to the survey. All centers used prophylaxis for the donor-positive/recipient-negative (D+/R-) group. For R + patients, 54% used prophylaxis, 37% used a pre-emptive strategy. In the low-risk group, D-/R-, 50% used a pre-emptive approach and 50% had no specific prevention strategy. The antiviral used by all centers for prophylaxis was valganciclovir (VGCV). The duration of prophylaxis varied from 3 to 7 months and the duration of viral load monitoring varied from 6 months to indefinitely. No center used a hybrid/sequential approach. For the treatment of CMV DNAemia, VGCV or intravenous GCV were used. Therapeutic drug monitoring of VGCV was performed in 5 centers (42%). Five centers reported drug resistance. Eight centers (67%) administered VGCV during the treatment of acute graft rejection.

**Conclusions:**

There is uniformity in CMV management in some areas among pediatric KT centers in France but not in others which remain diverse and are not up to date with current guidelines, suggesting unnecessary variation which could be reduced with better evidence to inform practice.

## Introduction

Kidney transplantation (KT) is considered the optimal treatment for children with end-stage kidney disease. Infections are a permanent risk in patients receiving immunosuppressive treatment and may impact graft survival. Cytomegalovirus (CMV) is the most common opportunistic pathogen infecting solid organ transplant recipients (1). It is widespread and is associated with increased mortality and morbidity (2). For this reason, CMV prevention strategies are commonly used in such patients. However, CMV infection and disease may still occur despite preventive therapies. The primary risk factor for CMV infection or disease is the CMV serostatus of the donor/recipient pair. CMV donor-positive/recipient-negative (D+/R-) patients are at the highest risk of developing CMV infection and disease through primary infection with the virus. CMV R + patients have an intermediate risk (reactivation). CMV D-/R- patients are at a low risk for CMV infection and disease (3).

Prevention and treatment of CMV infection and disease in pediatric and adolescent organ transplant recipients present several unique issues compared to the adult population. Indeed, children are more likely to be CMV-seronegative than adults, and KT from CMV-seropositive donors to CMV-negative recipients is more frequent. Patient recipients considered at a low-risk for CMV infection and disease (CMV D-/R-) are at risk of acquiring *de novo* CMV infection as a result of increased exposure in the community (school, young siblings, daycare facilities) (4). These data suggest that the epidemiology of CMV in the adult population may not be extrapolated to the pediatric population.

Two well-recognized clinical practice guidelines outlining CMV screening, prevention and treatment strategies in solid organ transplantation have been published and updated (1, 4). They summarized the existing evidence on CMV infection for all solid organ transplantations. However, despite the availability of these guidelines for both adults and children, evidence-based data is lacking in the field of pediatric KT and the optimal CMV prevention and management strategies remain unknown in this population. Current clinical practices can vary significantly between centers (2, 5, 6) which may be suboptimal for CMV prevention and management in pediatric kidney transplant recipients.

Our goal was to describe CMV prevention and management strategies in France with reference to the American Society of Transplantation (4) and the Transplantation Society International CMV Consensus Group guidelines (1), and to identify any potential barriers to their implementation. The next step would be to move towards standardization of clinical practice in order to better investigate CMV epidemiology and outcomes in pediatric real-world observational studies. We therefore conducted a survey aiming at assessing current CMV prevention and treatment strategies used among pediatric KT centers in France.

## Methods

### Survey design and development

A survey was developed and reviewed by the transplantation working group of the French Society for Pediatric Nephrology. A 44-question web-based survey (in French) was sent electronically to all 13 pediatric kidney transplantation centers in France in 2018 (www.dragnsurvey.com). All participating centers are part of a separate pediatric transplant program. The responders were all pediatric nephrologists and it was requested that only one respondent per center could reply. The survey is provided in the Supplementary Material. Responses were collected in 2019. The survey was composed of several sections focusing on different aspects of CMV prevention and treatment. A first section asked whether a local protocol was already in place. A series of items about which preventive strategy was used, the choice of antiviral, the length of surveillance, were requested according to the CMV serostatus of the donor and the recipient (i.e., D+/R-, R + irrespective of the donor, and D-/R-). The survey also focused on adjunctive therapies, therapeutic drug monitoring, acute graft rejection and the use of lymphocyte-depleting agents. Answers of partially completed surveys were included, although, where questions were unanswered, this was reflected in the denominators accordingly.

Glomerular filtration rate (eGFR) was estimated from the serum creatinine concentration measured by local laboratories, using the revised Schwartz equation (7). Body surface area (BSA) was calculated using Mosteller's equation: BSA (m²) = [height (cm) × weight (kg)/3600].

### Definitions

Survey participants were asked to use the following definitions that were specified at the start of the survey:

– Prophylaxis: antiviral medication for a specified time period. Prophylaxis can be universal (given to all recipients) or targeted (given based on risk profile to selected groups of recipients).
– Pre-emptive therapy: serial monitoring for CMV replication with the initiation of therapy at a predetermined threshold viral load.
– Sequential/hybrid therapy: short-course prophylaxis followed by serial monitoring and pre-emptive therapy as above.
– Active CMV infection: presence of CMV replication in the blood regardless of whether signs or symptoms are present.
– CMV disease: presence of viral replication in blood accompanied by clinical manifestations. CMV disease may manifest with either CMV syndrome or tissue-invasive CMV disease.
– CMV syndrome: presence of detectable viral replication in blood accompanied by attributable symptoms and signs (e.g., fever, malaise, arthralgia, leukopenia, thrombocytopenia) in the absence of tissue-invasive disease.
– Tissue-invasive CMV disease: presence of viral replication in blood with clinical symptoms and signs of end-organ disease (e.g., enteritis, colitis, hepatitis, pneumonitis, meningitis, encephalitis, retinitis).

## Results

Twelve out of 13 centers (92%) responded to the survey. All responders were pediatric nephrologists.

### Establishment of a protocol

Almost all centers had guidelines or protocols related to the prevention and treatment of CMV after KT (11 out of 12 centers) uniformly in place at each center. The survey did not explore whether the protocol was based on current clinical practice guidelines or not.

### Laboratory tests, CMV detection methods

Serologic assays were used in all centers for pre-transplant screening. The method of choice for post-transplant testing was CMV polymerase chain reaction (PCR) (reflecting the viral load or quantitative nucleic acid amplification testing). Ten (83%) centers used whole blood specimens and 2 (17%) centers used plasma specimens. All responders only used one method per center. No centers used urine CMV PCR, urine viral culture, nor CMV antigenemia for pre or post-transplant screening. Diagnostic of tissue-invasive disease were not discussed.

### Risk stratification strategy by donor–recipient CMV serostatus

The survey inquired about prevention strategies used according to the donor-recipient CMV serostatus. CMV prevention strategies were dominated by prophylaxis (Table 1). One center had no prevention strategy for the group R+.

**Table 1:** CMV prevention strategies: different strategies used in each centre according to donor and recipient serostatus (*n* = 12).

CMV serotype	Prophylaxis	Pre-emptive	Hybrid	No prevention strategy
D+/R-	12 (100%)	0	0	0
D+/R+	7 (58%)	4 (33.5%)	0	1 (8.5%)
D-/R+	6 (50%)	5 (41.7%)	0	1 (8.3%)
D-/R-	0	6 (50.0%)	0	6 (50.0%)

CMV, cytomegalovirus; D, donor; R, recipient.

### Prophylaxis

All responders (*n* = 12) used a prophylactic approach for the group D+/R-, 58% for the group D+/R+, 50% for the group D-/R + and 0% for the group D-/R- (Table 2). The most commonly used antiviral was valganciclovir (VGCV). One center used intravenous ganciclovir (GCV) during the initial post-transplant stage then transitioned to oral prophylaxis by VGCV.

**Table 2:** CMV prevention strategies: prophylaxis summary.

Centre	Serostatus	Medication (Route)	Dose (mg)	Duration (weeks)	Viral load monitoring (during prophylaxis)	Viral load monitoring (after prophylaxis)
1	D+/R-	VGCV (PO)	7 × BSA (m^2^) × GFR ml/min/1.73 m^2^ max 900 mg/day	24	M0–M3: every 2 weeks M3–M6: monthly	M6–M12: monthly
2	D+/R-	VGCV (PO)	900 mg/1.73 m^2^/day	26	Monthly	Weekly for 1 month Then monthly for 11 months
3	D+/R-	GCV (IV) + VGCV (PO)	GCV: 5 mg/kg/12 h while PIVC Adapted for GFR VGC: 7 × BSA (m^2^) × GFR ml/min/1.73 m^2^ max 900 mg/day	24	M0–M3: weekly M3–M6: every 2 weeks	M6–M24: monthly Then every 3 months indefinitely
4	D+/R- and R+	VGCV (PO)	7 × BSA (m^2^) × GFR ml/min/1.73 m^2^ max 900 mg/day	24	M0–M3: weekly M3–M6: every 2 weeks	M6–M18: every 2 weeks M18–M24: monthly
5	D+/R-	VGCV (PO)	7 × BSA (m^2^) × GFR ml/min/1.73 m^2^ max 900 mg/day	26	M0–M1: weekly M0–M6: monthly	Monthly indefinitely
6	D+/R- and R+	VGCV (PO)	7 × BSA (m^2^) × GFR ml/min/1.73 m^2^ max 900 mg/day	12	None	1 week and 1 month after the end of prophylaxis M2–M12: every 3 months Then ×1 per year indefinitely
7	D+/R-	VGCV (PO)	900 mg/1.73 m^2^/day	24	Monthly	M6–M12: monthly
8	D+/R- and R+	VGCV (PO)	900 mg/day adapted for GFR	24	Monthly	M6–M24: monthly
9	D+/R- R+	VGCV (PO)	7 × BSA (m^2^) × GFR ml/min/1.73 m^2^ max 900 mg/day	14	Weekly	M3–M6: every 2 weeks M6–M12: monthly M12–M24: every 2 months Then ×3 per year indefinitely
10	D+/R- and R+	VGCV (PO)	7 × BSA (m^2^) × GFR ml/min/1.73 m^2^ max 900 mg/day	24	None	M6–M12: every 2 weeks
11	D+/R- and R+	VGCV (PO)	7 × BSA (m^2^) × GFR ml/min/1.73 m^2^ max 900 mg/day	At least 14 weeks, 28,5 weeks if possible	M0–M3: weekly M3–M6: every 2 weeks	M6–M18: monthly Then every 3 months indefinitely
12	D+/R-	VGCV (PO)	15 mg/kg/day once a day (max 900 mg/day)	12	M0–M3: weekly	M3–M6: every 2 weeks, then at M8, M10, M12 Then every 3 months indefinitely

CMV, cytomegalovirus; D, donor; R, recipient; GCV, ganciclovir; VGCV, valganciclovir; IV, intravenous; PO, orally; BSA, body surface area; GFR, estimated glomerular filtration rate; Max, maximum; M0, month 0; PIVC, peripheral intravenous catheter; yrs, years of age.

Eight out of 12 centers (67%) used the following BSA-based dosing equation for VGCV: 7 × BSA [m²] × eGFR (Schwartz formula) [ml/min/1.73 m²], maximum 900 mg per day. The most frequent duration (75% of centers) for prophylaxis was 6–7 months (24–28 weeks). The remaining 25% adopted prophylaxis for 3 months. Eleven centers (92%) monitored CMV DNAemia during prophylaxis with an interval schedule varying from weekly to monthly. Viral load monitoring after prophylaxis varied considerably from 12 months to indefinitely.

### Pre-emptive therapy

Pre-emptive therapy was not reported for the subgroup at the highest risk of CMV DNAemia (D+/R-). In the R + group (i.e., D-/R + or D+/R+), 37.5% of centers used a pre-emptive approach (Table 1). Pre-emptive therapy was used by 50% of centers for the low-risk group (D-/R-).

In D-/R + and D+/R + patients, a pre-emptive strategy was applied in 42% (*n* = 5) and 33% (*n* = 4) of KT centers, respectively. The frequency of monitoring immediately after KT varied: weekly (*n* = 7 centers), every 2 weeks (*n* = 1), every 4 weeks (*n* = 3), and then the frequency of monitoring was reduced after 3 to 6 months. The ultimate duration of viral monitoring when using a pre-emptive therapy approach varied considerably from 12 months (*n* = 4), 24 months (*n* = 2) to indefinitely (*n* = 5). Only one center never used a pre-emptive approach for any of the groups.

Intervention thresholds varied and were not all reported: as soon as detected (*n* = 2), 500 IU/L in whole blood (*n* = 1), 206.4 copies/ml in plasma sample (*n* = 1) or 500 copies/ml in whole blood (*n* = 2), 2000–5000 copies/ml (*n* = 1). Four centers used a logarithmic scale with variable thresholds: >3 log IU/mL, 2–3 log copies/mL (*n* = 1), 4 log copies/ml (*n* = 2). If CMV replication was detected and asymptomatic it was treated using VGCV in 100% of centers.

### Hybrid/sequential therapy

None of the responders used a hybrid/sequential approach.

### CMV infection treatment

For the treatment of CMV infection, the majority of centers used oral VGCV (58%, *n* = 7), most often at 2×[7 × BSA × eGFR], maximum 900 mg twice a day. Five out of 7 centers giving VGCV as a first-line treatment, used this initial dose for 3 weeks, followed by a prophylactic dose, provided clearance of CMV viremia, for the following 1 to 3 months [i.e., 7 × BSA × eGFR], maximum 900 mg/day. Other centers used a first-line treatment by intravenous GCV (42%, *n* = 5), at 5 mg/kg/12 h adapted to GFR for 15–21 days. One center considered a switch to oral VCGV after 3 weeks of treatment with GCV.

### Strategy in patients receiving lymphocyte depleting agents

Alterations in CMV prevention strategies were rarely reported for patients who received anti-thymocyte globulin either for induction therapy or treatment for acute cellular rejection. Most centers (*n* = 9, 75%) did not change their strategy when a lymphocyte depleting agent (anti-thymocyte globulin) was used.

### Prevention during treatment of acute rejection

Eight centers (67%) administered CMV prophylaxis with VGCV (7 × BSA × GFR), maximum 900 mg/day or 15 mg/kg/day max 900 mg/day) during treatment of acute rejection. The duration of this treatment varied from 3 to 6 months or throughout corticosteroid therapy.

### Adjunctive therapies

No center reported the administration of CMV specific Immunoglobulin. Two centers reported the administration of 1–2 infusions of intravenous immunoglobulin (0.4 g/Kg) when treating CMV infection depending on the CMV viral load, the IgG count and the severity of the disease.

### Therapeutic drug monitoring

Therapeutic drug monitoring (TDM) was used in 42% (*n* = 5) of centers, for GCV (*n* = 2), for VGCV (*n* = 1), for VGCV and GCV (*n* = 2). Systematic TDM was only used in one center on day 5 of CMV treatment (GCV and VGCV). The other centers used TDM when drug resistance or non-adherence to treatment were suspected.

### Drug resistance

Five centers (42%) reported drug resistance. Two centers reported drug resistance for GCV and two for VGCV. Only one center identified 2 mutations using genome sequencing (UL97 mutation, resistance C603W and UL97 mutation, polymorphism 919 E N68 D S108 N I244 V). One center suspected drug resistance and subsequently treated the patient with Maribavir. Foscarnet (FOS) was prescribed in 3 centers when drug resistance to the first-line treatment was probable (60 mg/kg per 8 h). The duration of second-line treatment with FOS was specified in two centers: treatment for 3 weeks in one center and until negative CMV PCR in another. One center reported clinically drug resistance to VGCV but no mutation and used GCV (double dose) for 3 weeks with a favorable outcome.

## Discussion

The goal of this study was to investigate recent practices and determine differences in CMV prevention and treatment strategies among French pediatric KT centers in the current era of more specific diagnostic testing, alternatives to universal prophylaxis and newer antiviral therapies. The findings provide a real-world snapshot, covering almost all French pediatric KT centers. This multicenter survey shows interinstitutional variability which reflects previously known uncertainties regarding CMV prevention and treatment strategies for children. Certain aspects of the prevention and management of CMV infection reported in this survey diverged from current guidelines (Table 3). It is difficult to determine the reasons for these discrepancies. A large survey from the Working Group of the European Society for Organ Transplantation (adults) noted similar discrepancies in the management of CMV and suggested that may be related to budgetary or reimbursement policies in specific countries (8). However, our survey showed very similar results and a non-uniform approach despite France having the same reimbursement policy for all children and limited budgetary limitations, especially for medications recommended by international guidelines. These guidelines insist on the fact that there are considerably fewer pediatric studies to support current recommendations for CMV prevention and treatment that are extrapolated from studies conducted in adults, this may contribute to the differences observed in our study. Fortunately, our survey revealed very few practices that were clearly not recommendable, based on current evidence (1, 4).

**Table 3:** Discrepancies between survey-reported practices and specific pediatric guideline recommendations*

	Survey-reported practice	Guideline recommandation
Pre-KT screening	All centers used serological assays for all patients 25% of centers also performed CMV QNAT (blood)	In pediatric SOT candidates <18 months of age who may have passively acquired maternal CMV IgG antibody, CMV QNAT or culture of urine specimen may be performed to determine baseline CMV status.
CMV prophylaxis prescription	For intermediate-risk patients (R+): 17% applied no preventative strategy	For intermediate-risk patients (R+): Prophylaxis or Preemptive Alternate strategy: Hybrid approach
VGCV dosing strategy	67% used the following BSA-based dosing equation for VGCV: 7 × BSA [m²] × eGFR (Schwartz formula) [ml/min/1.73 m²], maximum 900 mg per day	Dosing of VGCV in children should be based on BSA, renal function over the prior suggestion of 15–16 mg/kg dosing
Prevention in patients receiving lymphocyte depleting agents and during acute rejection	75% did not change their strategy (no preventive approach) when a lymphocyte depleting agent (anti-thymocyte globulin) was used	In children at risk for CMV who receive significantly intensified immunosuppression (eg, antilymphocyte therapy, intravenous steroids) for rejection, primary disease recurrence, or other complicating condition, we recommend either prophylaxis with (val)ganciclovir or preemptive therapy
	33% did not apply either CMV prophylaxis, nor a preemptive strategy during treatment of acute rejection	
Duration of CMV monitoring post prophylaxis	Viral load monitoring after prophylaxis varied considerably from 12 months to indefinitely.	The risk of post-prophylaxis delayed-onset CMV disease is highest during the first 3 months after cessation of antiviral prophylaxis, and pediatric SOT patients should undergo CMV QNAT surveillance during this at-risk period.
Duration of CMV monitoring during preemptive approach	Viral load monitoring when using a pre-emptive therapy approach varied considerably from 12 months (33%), 24 months (17%) to indefinitely (*n* = 42%).	For pediatric patients undergoing the strategy of preemptive therapy, CMV QNAT is recommended once weekly for at least 12 weeks.

BSA, body surface area; CMV, cytomegalovirus; KT, kidney transplantation; QNAT, quantitative nucleic acid amplification test; R, recipient; SOT, solid organ transplant; VGCV, valganciclovir.

*Guidelines of the American society of transplantation infectious disease community of practice and the third international consensus guidelines on the management of cytomegalovirus in solid-organ transplantation.

Regarding CMV prevention, strategies were determined according to donor recipient serostatus as in current guidelines. All centers adopted prophylaxis for high-risk recipients, strategies used for intermediate-risk recipients varied significantly (prophylaxis/preemptive/clinical surveillance) and centers used either clinical surveillance or pre-emptive therapy for the low-risk group.

The medication of choice for CMV prophylaxis was oral VGCV, however VGCV dosing strategies varied considerably between centers. Recent guidelines recommend the use of the VGCV-dosing algorithm that adjusts for BSA and renal function using the updated Schwartz formula (1, 4). A survey in the United States in 2017 found that less than 60% of the survey respondents used the FDA approved dosing equation (7 x BSA x eGFR) for VGCV in CMV prophylaxis (5). Our survey showed similar results with 67% of centers who used the recommended VGCV-dosing equation. This highlights the need for more clinical and pharmacokinetic data surrounding VGCV use in pediatric patients to allow for an international consensus on an optimal dosing strategy and thus to determine a possible correlation between dosing method and clinical outcomes.

Regarding treatment of CMV infection, this survey highlighted that pediatric KT centers have largely implemented the recently updated guidelines regarding the use of oral VGCV for treatment of CMV infection with the majority of centers using VGCV as the first line of treatment (1, 4, 9). This most likely reflects pediatric nephrologist's wish to avoid complications and the burden of prolonged intrevenous GCV treatment (central venous catheter, prolonged hospital admission). However, the guidelines remain clear that IV GCV should be used for severe CMV disease (1). Acyclovir (ACV), valacyclovir (VACV), intravenous GCV, oral GCV, and VGCV have all been studied for universal prophylaxis. An adult study (randomized, open-label, single-center trial), concluded that compared with VACV prophylaxis, pre-emptive VGCV therapy may lead to less severe interstitial fibrosis and tubular atrophy and to significantly better graft survival (10).

No pediatric trial has compared different CMV prevention strategies. However, Suresh et al. suggested that a risk-stratified approach using a hybrid or prophylactic strategy for D+/R- patients, a pre-emptive strategy for R+, and clinical follow-up alone for D-/R- patients resulted in very low rates of CMV disease with minimal adverse effects (11). In our survey, we found a low uptake for preemptive strategies especially in the high-risk group, despite this strategy having been approved as an acceptable alternative to prophylaxis in current guidelines (3). Another pediatric survey, but in liver transplant patients, also reported a low use of preemptive strategy in high-risk patients, with preemptive therapy reported by only 2 sites out of 29 as primary CMV prevention strategy in this group (6). The reasons behind this are unclear, but we can hypothesize that a preemptive approach is more difficult to coordinate and viral load thresholds are not defined leading to possible lack of clarity in the implementation of the strategy and as a physician, prophylaxis may be regarded as an easier or a safer option. No hybrid approach has been described in pediatric KT. A study including pediatric liver and heart transplant recipients suggested that a hybrid preventative approach for CMV was a reasonable alternative to prolonged antiviral prophylaxis and may reduce unnecessary exposure to antiviral therapy (12, 13).

The duration of prophylaxis in this survey was variable and ranged from 12 weeks to 28 weeks (200 days). An international, randomized, prospective, double-blind study, compared 318 CMV D+/R- adult KT recipients receiving 900 mg VGCV once daily for up to 200 days vs. 100 days and showed that extending VGCV prophylaxis from 100 to 200 days was associated with a sustained reduction in CMV disease up to 2 years post-transplant but no improvement in rejection rate and graft survival (14). A pediatric KT retrospective study assessing the efficacy of an extended CMV prophylaxis from 6 months to 12 months concluded that it was safe and had minimal adverse effect, but it did not reduce CMV infection or disease (15).

In adults, routine monitoring for CMV DNAemia during prophylaxis is not recommended. However, in the pediatric population, some experts advocate surveillance during prophylaxis due to concern for breakthrough DNAemia (1). Duration of monitoring for CMV DNAemia after prophylaxis varied greatly between centers from 6 months to indefinitely. Little data is available on the optimal duration of viral surveillance. Use of surveillance after prophylaxis may be considered in patients at increased risk for post-prophylaxis CMV disease. Based on the experience with pre-emptive therapy, the value is probably greatest if done weekly for at least 12 weeks after the end of prophylaxis (1).

The threshold for intervention when adopting a pre-emptive approach remains to be defined. Thresholds differ between recent research studies and often depended on the risk of CMV replication. Further studies are needed to determine the consensus threshold in IU/ml (1). None of the responders adopted a hybrid approach. This approach limits the duration of prophylaxis to the period of most intense immunosuppression. Previous surveys concerning CMV management practices in pediatric lung and liver transplant observed a higher uptake of a hybrid strategy (6, 16). However, current guidelines state that a hybrid approach for pediatric KT is only recommended as an alternative to prophylaxis or a pre-emptive strategy for intermediate-risk patients (1), thus reflecting the lack of data in pediatric KT.

TDM was only used in 42% of centers, although it might be important for the management of CMV infection or disease. Suboptimal dosing may increase the risk for clinical treatment failure and the development of resistance, while supratherapeutic doses may increase toxicity (1). The exact role of TDM for the management and drug resistance is not fully established which is reflected by the limited uptake in our survey.

The burden of CMV in pediatric and kidney transplant is difficult to establish and is based on a limited number of pediatric cohort studies (11). The incidence of CMV DNAemia after pediatric KT is approximately 20%, with disease in 1%–10% (1), but can vary greatly between studies. A retrospective study of the CERTAIN Registry analyzed the epidemiology of CMV in a cohort of 242 pediatric KT recipients and found that CMV replication was associated with a more pronounced decline of graft function at 3 year post-transplant (2), results that were similar to what has been found in adult transplant recipients (17). In addition, the use of antiviral prophylaxis in CMV D+/R- and D+/R + patients was accompanied by a higher CMV-free survival and a lower decline in eGFR compared with preemptive therapy (2). Another study demonstrated an association between subclinical CMV infection, which occurred despite standard antiviral prophylaxis, and chronic allograft injury in pediatric KT recipients (18). Studies tend to show that CMV prophylaxis delays rather than prevents CMV DNAemia (1). These studies outlining the burden of CMV after KT highlight the need for the development of robust, evidence-based guidelines in attempt to reduce this significant burden and improve clinical outcomes in pediatric KT.

Limitations to the study design exist. Missing information or incomplete answers were addressed by targeted emails, but certain missing data remained. In addition, although one response per center was requested, the team member surveyed at each center may not necessarily represent practice in that center. However, the survey tried to mitigate this risk by inquiring about the use of protocols in each center, which was the case in over 90% of responding centers. It is unclear whether respondents were aware of recent guidelines and unfortunately, our study did not explore the reasons for deviation from guidelines. Moreover, there is no information about clinical outcomes such as rates of CMV infection and disease at each center or graft loss due to CMV, only practice patterns. Our results derive from a survey and do not use patient data which would give a more insightful picture in order to assess if a particular practice pattern impact the outcomes of CMV. However, our provide data for future studies to evaluate the burden of CMV in pediatric KT recipients.

## Conclusion

There is uniformity in CMV management in some areas among pediatric KT centers in France but other areas of practice remain diverse and were not necessarily up to date with current guidelines, suggesting unnecessary variation which could be reduced with better evidence to inform practice. Standardization of practices allow a better appreciation of the burden of CMV in this population as non-uniform approaches hamper data interpretation. Data on CMV infection and management in pediatric KT patients should be routinely collected as part of prospective trials to inform guidelines and improve prevention and treatment of this important complication. These findings may help to design studies to evaluate safety and efficacy of new strategies to prevent and treat CMV infection, as well as comparing clinical outcomes according to these strategies in pediatric KT recipients.

## Data Availability

The original contributions presented in the study are included in the article/**Supplementary Material**, further inquiries can be directed to the corresponding author/s.
