# Supplementary material for: National survey of prevention and management of CMV infection in pediatric kidney transplantation in comparison to clinical practice guidelines
Source: Front Pediatr. 2022 Dec 16;10:1057352. doi: 10.3389/fped.2022.1057352 (PMC9800817; doi:10.3389/fped.2022.1057352)
Supplement: Supplementary file 1 [file Datasheet1.docx]

**Supplementary data online 1:**

**Table S1. Demographics of French pediatric transplant centers in 2019**

| Center | Number of KT performed in 2019 | | Number of KT patients followed-up per center* | Number of pediatric nephrologists (not counted as FTE) | Urban vs Rural location |
| --- | --- | --- | --- | --- | --- |
| Bordeaux | | 8 | 34 | 4 | Mainly rural |
| Lille | | 7 | 36 | 5 | Mainly urban |
| Lyon | | 15 | 102 | 6 | Mainly urban |
| Marseille | | 6 | 39 | 5 | Mainly urban |
| Montpellier | | 3 | 21 | 4 | Mainly rural |
| Nancy | | 2 | 8 | 3 | Mainly rural |
| Nantes | | 12 | 66 | 4 | Mainly rural |
| Paris Necker | | 14 | 91 | 6 | Mainly urban |
| Paris Robert Debré | | 21 | 88 | 7 | Mainly urban |
| Strasbourg | | 4 | 12 | 3 | Mainly rural |
| Toulouse | | 4 | 26 | 5 | Mainly rural |
| Tours | | 3 | 16 | 3 | Mainly rural |

*Estimation of the number of patients with a functioning graft followed-up per center

KT, kidney transplantation; FTE, full time equivalent

**Supplementary data online 2:**

**Web-based Survey**

The purpose of this survey is to assess the breadth of Cytomegalovirus (CMV) prevention and treatment strategies across French paediatric kidney transplant centres. This survey will be sent to every paediatric kidney transplant centre in France.

Only one survey per participating centre should be completed.

The time needed to fill in this survey is around 15 minutes.

Thank you for your help.

1. First and last name:

Email address:

Institution:

Comments:

1. Does your current centre have a protocol regarding CMV prevention post paediatric kidney transplantation?
2. Yes
3. Non
4. Elaboration in process
5. I don’t know

**Regarding CMV detection methods:**

1. Which detection method does your centre use for CMV screening pre-kidney transplant?
2. CMV serology
3. PCR CMV blood
4. PCR CMV urine
5. CMV viral culture urine
6. CMV antigenemia
7. None of the above, specify: …
8. Which CMV detection method does your centre use after kidney transplantation?
9. PCR CMV Whole blood
10. PCR CMV Plasma
11. CMV pp65 antigenemia
12. CMV pp67 antigenemia
13. Other CMV antigenemia, specify: …

Regarding CMV prevention strategies:

**Please use the following definitions:**

**- Prophylaxis:** antiviral medication for a specified period of time. Prophylaxis can be universal (given to all recipients) or targeted (given based on risk profile to selected groups of recipients).

**- Pre-emptive therapy:** serial monitoring for CMV replication with initiation of therapy at a predetermined threshold viral load.

**- Sequential/hybrid therapy:** short-course prophylaxis followed by serial monitoring and pre-emptive therapy as above.

**- Active CMV infection**: presence of CMV replication in the blood regardless of whether signs or symptoms are present.

**- CMV disease:** presence of viral replication in blood accompanied by clinical manifestations. CMV disease may manifest with either CMV syndrome or tissue-invasive CMV disease.

- CMV syndrome: presence of detectable viral replication in blood accompanied by attributable symptoms and signs (e.g., fever, malaise, arthralgia, leukopenia, thrombocytopenia) in the absence of tissue-invasive disease.
- Tissue-invasive CMV disease: presence of viral replication in blood with clinical symptoms and signs of end-organ disease (e.g., enteritis, colitis, hepatitis, pneumonitis, meningitis, encephalitis, retinitis).

**Regarding the group Donor CMV positive (D+) and Recipient CMV negative (R-):**

1. Which strategy does your centre use for the prevention of CMV for D+/R- patients?
2. Prophylaxis
3. Pre-emptive therapy
4. Hybrid/Sequential therapy
5. None
6. For prophylaxis, which is the first antiviral used?
7. Acyclovir
8. Valacyclovir
9. Ganciclovir
10. Valganciclovir
11. Not relevant
12. Other, specify: …
13. For prophylaxis, what and how is the dose administered?
14. Dose: …
15. Administration: …
16. Not relevant
17. What is the length of treatment, in weeks?
18. Treatment length: …
19. Not relevant

**Example of an answer for questions 9,10 and 13:**

How frequently are the patients monitored…?

Month 0 – Month 3: weekly

Then from M3-M6: every 15 days

Then at M8, M10 and M12

Then every 3 months for a year

1. How frequently are patients monitored DURING prophylaxis?

Free text: …

1. How frequently are the patients monitored AFTER prophylaxis?

Free text: …

1. For prophylaxis, how long are patients monitored for, in months, after transplantation?
2. 3 months
3. 6 months
4. 12 months
5. 24 months
6. Not relevant
7. Other, specify: …
8. For pre-emptive therapy, what is the viral threshold for intervention? (Please include unit of measure copies/ml, IU/ml, antigen positive cells, etc…)
9. Threshold: …
10. Not relevant
11. For pre-emptive therapy, how frequently are patients monitored after transplantation?

Free text: …

1. For pre-emptive therapy, how long are patients monitored for, in months, after transplantation?
2. 2 months
3. 3 months
4. 6 months
5. 12 months
6. 24 months
7. Not relevant
8. Other, specify: …
9. For the treatment of active CMV infection, which is the first antiviral used? What dose? For how long?
10. Antiviral: …
11. Dose with unit: …
12. Treatment length, in weeks:…
13. Not relevant
14. Comments: …

**Regarding the group (D+ or D-)/R+:**

1. Which strategy does your centre use for the prevention of CMV for R+ patients?

a. Prophylaxis

b. Pre-emptive therapy

c. Hybrid/Sequential therapy

d. None

1. For prophylaxis, which is the first antiviral used?
2. Acyclovir
3. Valacyclovir
4. Ganciclovir
5. Valganciclovir
6. Not relevant
7. Other, specify: …
8. For prophylaxis, what and how is the dose administered?
9. Dose: …
10. Administration: …
11. Not relevant
12. What is the length of treatment, in weeks?
13. Treatment length: …
14. Not relevant

**Example of an answer for questions 20, 21 and 24:**

How frequently are the patients monitored…?

Month 0 – Month 3: weekly

Then from M3-M6: every 15 days

Then at M8, M10 and M12

Then every 3 months for a year

1. How frequently are patients monitored DURING prophylaxis?

Free text: …

1. How frequently are the patients monitored AFTER prophylaxis?

Free text: …

1. For prophylaxis, how long are patients monitored for, in months, after transplantation?
2. 3 months
3. 6 months
4. 12 months
5. 24 months
6. Not relevant
7. Other, specify: …
8. For pre-emptive therapy, what is the viral threshold for intervention? (Please include unit of measure copies/ml, IU/ml, antigen positive cells, etc…)
9. Threshold: …
10. Not relevant
11. For pre-emptive therapy, how frequently are patients monitored after transplantation?

Free text: …

1. For pre-emptive therapy, how long are patients monitored for, in months, after transplantation?
2. 2 months
3. 3 months
4. 6 months
5. 12 months
6. 24 months
7. Not relevant
8. Other, specify: …
9. For the treatment of active CMV infection, which is the first antiviral used? What dose? For how long?
10. Antiviral: …
11. Dose with unit: …
12. Treatment length, in weeks: …
13. Not relevant
14. Comments: …

**Regarding the group D-/R-:**

1. Which strategy does your centre use for the prevention of CMV for D-/R- patients?

a. Prophylaxis

b. Pre-emptive therapy

c. Hybrid/Sequential therapy

d. None

1. For prophylaxis, which is the first antiviral used?
2. Acyclovir
3. Valacyclovir
4. Ganciclovir
5. Valganciclovir
6. Not relevant
7. Other, specify: …
8. For prophylaxis, what and how is the dose administered?
9. Dose: …
10. Administration: …
11. Not relevant
12. What is the length of treatment, in weeks?
13. Treatment length: …
14. Not relevant

**Example of an answer for questions 31, 32 and 35:**

How frequently are the patients monitored…?

Month 0 – Month 3: weekly

Then from M3-M6: every 15 days

Then at M8, M10 and M12

Then every 3 months for a year

1. How frequently are patients monitored DURING prophylaxis?

Free text: …

1. How frequently are the patients monitored AFTER prophylaxis?

Free text: …

1. For prophylaxis, how long are patients monitored for, in months, after transplantation?
2. 3 months
3. 6 months
4. 12 months
5. 24 months
6. Not relevant
7. Other, specify: …
8. For pre-emptive therapy, what is the viral threshold for intervention? (Please include unit of measure copies/ml, IU/ml, antigen positive cells, etc…)
9. Threshold: …
10. Not relevant
11. For pre-emptive therapy, how frequently are patients monitored after transplantation?

Free text: …

1. For pre-emptive therapy, how long are patients monitored for, in months, after transplantation?
2. 2 months
3. 3 months
4. 6 months
5. 12 months
6. 24 months
7. Not relevant
8. Other, specify: …
9. For the treatment of active CMV infection, which is the first antiviral used? What dose? For how long?
10. Antiviral: …
11. Dose with unit: …
12. Treatment length, in weeks: …
13. Not relevant
14. Comments: …

Regarding all post kidney transplant patients:

1. Does your centre modify their CMV prevention practices if thyroglobulin induction therapy is used?
   1. No, never
   2. Yes, for the group D+/R-
   3. Yes, for the group R+
   4. Yes, for the group R-/D-
   5. Comments: …
2. When treating active CMV infection, do patients receive an immunoglobulin adjunctive therapy?
   1. Yes
   2. No
   3. If yes, intravenous immunoglobulin
   4. If yes, CMV immunoglobulin
3. Regarding immunoglobulin adjunctive therapy:
   1. How frequently is it administered? …
   2. At what dose (please provide unit)? …
   3. For which group (D+/R-; R+; D-/R-)? …
   4. Not relevant
4. Does your centre use antiviral therapeutic drug monitoring?
   1. Yes
   2. No
5. Regarding therapeutic drug monitoring:
   1. For which antiviral? …
   2. At which point during treatment? …
   3. What are your target drug levels? …
   4. Not relevant
6. Does your centre (re)start CMV prophylaxis for patients with acute rejection (cell or antibody mediated rejection)?
   1. Yes
   2. No
7. Regarding CMV prophylaxis and acute rejection:
   1. What antiviral is used?
   2. At what dose?
   3. For how long, in weeks?
   4. Not relevant
